# Co-transcriptional folding is encoded within RNA genes

**DOI:** 10.1186/1471-2199-5-10

**Published:** 2004-08-06

**Authors:** Irmtraud M Meyer, István Miklós

**Affiliations:** 1 Oxford Centre for Gene Function, University of Oxford, South Parks Road, Oxford OX1 3QB, UK; 2 Eötvös University and Hungarian Academic of Science, Theoretical Biology and Ecology Group, Pázmány Péter sétány 1/c, 1117 Budapest, Hungary

## Abstract

**Background:**

Most of the existing RNA structure prediction programs fold a completely synthesized RNA molecule. However, within the cell, RNA molecules emerge sequentially during the directed process of transcription. Dedicated experiments with individual RNA molecules have shown that RNA folds while it is being transcribed and that its correct folding can also depend on the proper speed of transcription.

**Methods:**

The main aim of this work is to study if and how co-transcriptional folding is encoded within the primary and secondary structure of RNA genes. In order to achieve this, we study the known primary and secondary structures of a comprehensive data set of 361 RNA genes as well as a set of 48 RNA sequences that are known to differ from the originally transcribed sequence units. We detect co-transcriptional folding by defining two measures of directedness which quantify the extend of asymmetry between alternative helices that lie 5' and those that lie 3' of the known helices with which they compete.

**Results:**

We show with statistical significance that co-transcriptional folding strongly influences RNA sequences in two ways: (1) alternative helices that would compete with the formation of the functional structure during co-transcriptional folding are suppressed and (2) the formation of transient structures which may serve as guidelines for the co-transcriptional folding pathway is encouraged.

**Conclusions:**

These findings have a number of implications for RNA secondary structure prediction methods and the detection of RNA genes.

## Background

Most of the existing computational methods for RNA secondary structure prediction fold an already completely synthesized RNA molecule. This is done either by minimizing its free energy (e.g. done by MFOLD [1-3] and by the programs of the VIENNA package [4-8]) or by maximizing the probability under a model whose parameters can incorporate a variety of different sources of information, e.g. comparative information, free energy and evolutionary information (e.g. [9], TRNASCAN-SE [10], PFOLD [11,12] and QRNA [13]). All of these programs, including those that predict folding pathways by folding an already synthesized RNA sequence [14,15], therefore disregard the effects that co-transcriptional folding may have on the RNA's functional secondary structure. They essentially aim to predict the *thermodynamic RNA structure*, i.e. the secondary structure that minimizes the free energy of the molecule. However, theoretical studies of RNA molecules [16] indicate that the thermodynamic structure of even moderately long RNA molecules need not necessarily correspond to the *functional structure *which confers the desired functionality within the organism to the RNA molecule.

RNA molecules are known to fold as they emerge during transcription [17,18]. Transcription is a directed process of variable speed, during which the 5' end of the RNA molecule is synthesized before its 3' end. Hydrogen-bonds at the 5' end of the RNA molecule can thus form earlier in time than hydrogen-bonds involving the 3' end of the molecule. The thus emerging secondary structure elements can be transient or not, depending on their stability, their formation times and the availability and stability of competing alternative pairing partners. The directedness and also the speed of transcription can influence both the folding pathway and the functional secondary structure of the RNA molecule. We call this phenomenon *sequential *or *co-transcriptional folding *and call the resulting secondary structure the *kinetic structure *of the RNA molecule.

Co-transcriptional folding leads to the formation of temporary secondary structure elements [18,19]. The time that it takes to form and replace these transitory structure elements may successively narrow down the set of accessible folding pathways and may thereby guide the folding towards an ensemble of secondary structures which contains the desired functional secondary structure. However, these temporary secondary structure elements can also have distinct biological functions, e.g. in viroids [19] and as initial sites for protein anchoring during pre-mRNA transcription [20]. Based on experimental and theoretical investigations, Harlepp et. al. [21] and Isambert et. al. [22] found that temporary structures may form during transcription. All these results suggest that temporary secondary structure elements may play an important role in the correct folding of RNA sequences.

The speed of transcription also has an effect on folding which can be investigated by varying the nucleoside triphosphate concentration [19] or by transcribing RNA genes with viral polymerase T7 which has faster elongation during transcription than bacterial polymerases [23,24]. Both decreasing and increasing the natural speed of transcription can yield inactive transcripts [23,24]. Recent *in vitro *investigations of the *Tetrahymena *ribozyme [25] show that its co-transcriptional folding *in vitro *is twice as fast as the refolding of the entire RNA molecule under the same conditions and that both lead to the same functional folding. Moreover, they find that the co-transcriptional folding *in vitro *is still much slower than *in vivo.*

Among the multitude of biochemical processes which are known to occur transcriptionally [26,27], some processes act in order to prevent the mis-folding of RNA molecules. RNA chaperones are proteins which are believed to help refold mis-folded RNA structures by promoting intermolecular RNA-RNA annealing through non-specific interaction [28]. Without RNA chaperones, moderately long GC-rich helices have dissociation half-times of up to 100 years [29]. This time can be significantly reduced by RNA chaperones, which preferentially bind stretches of unfolded RNA and thereby decrease the kinetic barrier between the correct and incorrect secondary structure elements [28]. Specific RNA-binding proteins are also known to promote RNA folding by either guiding its folding or stabilizing its correct structure [30,31]. The hnRNP proteins non-specifically bind pre-messenger RNA and help in the splicing process [32].

RNA sequences can also promote the proper folding of other RNA sequences. It is known, for example, that the temporary interaction with highly conserved leader sequences of bacterial rRNA-operons is needed for the proper formation of 30S ribosomal subunits and the maturation of 16S rRNA [33,34].

All these experimental and the few theoretical findings suggest that co-transcriptional folding may play an important role in the correct folding of RNA molecules. They also show that the functional structure may only be a transient one which is available during a certain time span and that the functional structure need not correspond to the structure which would dominate the ensemble of structures after an infinite time span.

Little is known whether co-transcriptional folding is mainly governed by the specific or non-specific binding of proteins (or other molecules) which target the emerging RNA or whether the primary structure of the RNA molecule itself conveys the desired properties to guide its own correct co-transcriptional folding.

In this paper, we propose several statistics in order to detect, if and how co-transcriptional folding influences RNA sequences. Using these statistics, we show that the effects of co-transcriptional folding are widespread in RNA genes.

## Methods

### Theory

We want to show that an RNA sequence is organized in such a way to help the formation of the functional secondary structure during transcription. We aim to support this hypothesis by detecting two different features:

• **Possible competitors of helices in the functional structure are suppressed. **When the 3' end of a helix that is part of the final secondary structure emerges during transcription, the number of possible competitors for the 5' part of the helix should be as low as possible in order to promote the formation of the correct helix.

• **The folding pathway is engineered. **During transcription, several temporary helices are formed which may guide the folding process.

We investigate these features using several statistics which are based on the known primary and secondary structures of our RNA sequences. A crucial point in investigating these features is to define a set of statistics that have expectation of zero in the *H*_0 _case, when we suppose no co-transcriptional folding. However, verifying that these statistics have an expectation value of zero in the *H*_0 _case cannot simply be achieved by analyzing random sequences. Indeed, even generating random sequences is not trivial. First, it is hard to reliably predict the minimum free energy structure for the randomized sequences as most secondary structure prediction algorithms discard pseudo-knots and, even without pseudo-knots, predict only on average about 70 % of the base-pairs correctly. In addition, there is no guarantee that the secondary structure with the lowest free energy would correspond to the functional one. Second, even if the random sequences are generated by a shuffling algorithm which keeps the given secondary structure fixed, it cannot be guaranteed that the fixed structure remains the correct one for the new primary sequence. Generating random sequences therefore provides no straightforward solution for obtaining a *H*_0 _statistics with expectation value zero.

We circumvent this problem by studying pairs of statistics, where both statistics have the same, unknown expectation value in the *H*_0 _case and where one statistics has a bias away from the *H*_0 _expectation value in case of co-transcriptional folding, while the other statistics is not affected by co-transcriptional folding. By studying the difference of these two statistics, we thus gain a new statistics with expectation value zero in the case of no co-transcriptional folding and an expectation value larger or smaller than zero in the case of co-transcriptional folding.

**Figure 1:**
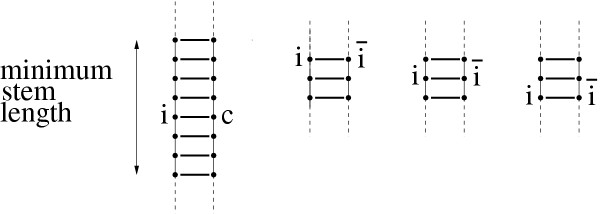
**Definition of a competing, alternative helix. **Pictorial definition of a competing, alternative helix. The known base-pair between sequence positions *i *and  has to have at least two other directly adjacent base-pairs within the known secondary structure (right) and the competing, alternative helix has to contain an alternative base-pair between sequence positions *i *and *c *(*c *is the competitor of ) which has to be contained within a helix of minimum stem length (left).

**Figure 2:**
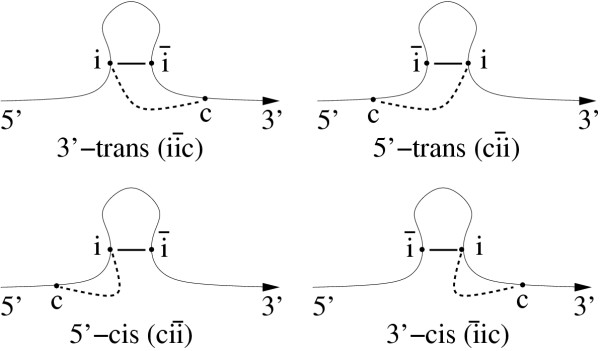
**Definition of the statistics. **Pictorial definitions of the four configurations 3'*cis*, 3'*trans*, 5'*cis *and 5'*trans *which correspond to the four statistics used to measure the directedness of RNA folding. Sequence positions *i *and  form a base-pair within the known secondary structure. Sequence position *c *is an alternative base-pairing partner for *i *(but according to the base-pairing rules therefore not for ) within a competing, alternative helix of a minimum length *min*_*stem*_. See the text for more explanation.

The statistics (which we will define in detail below) measure the presence of alternative helices which compete for at least one base-pair with the helices of the known secondary structure. These competing alternative helices are required to consists of at least *min*_*stem *_= 9 consecutive base-pairs of type {G - C, C - G, A - U, U - A, G - U, U - G} and are calculated by a dynamic programming procedure in which the known primary and secondary structure of the RNA is fixed, see Figure 1 for the definition of a competing, alternative helix. We checked that we obtain qualitatively similar results for smaller and larger *min*_*stem *_values (data not shown). While calculating all helices of at least *min*_*stem *_length, we test which of these helices constitute competing alternatives to helices of the known secondary structure and record each such competing case in one of our statistics. These alternative helices may be part of a pseudo-knotted structure and we do not discard them. As each of the two bases *i *and  of a base-pair in a known helix can have a competing alternative base-pairing partner within an alternative helix and as this alternative partner can either be found 5' (before), 3' (behind) or between the two strands of the known helix, all cases can be classified into six different classes. Of these six, we discard the two classes where the alternative helix falls between the two strands of the known helix as this un-paired loop region is typically too short to accommodate an alternative helix of at least *min*_*stem *_length. The remaining four classes, see Figure 2, can be sub-divided into two *cis- *and two *trans- *alternative classes, depending on whether the known base-pairing partners lie between the alternative base-pairing partners *(trans) *or not *(cis). *The four statistics 3'*cis, 3'trans, 5'cis *and *5'trans *that we use correspond to these four classes.

It is important to note that even without co-transcriptional folding, the destabilizing effects of competing *cis- *and *trans*-alternative helices are not necessarily the same as the stacking energies are not symmetric with respect to the 5' → 3' direction of the RNA sequence [3]. In addition, alternative *cis*-pairing partners are closer to the known pairing partners than *trans*-pairing partners and may thus lead more easily to incorrect helices. We may therefore compare only *cis*-competitors with other *cis*-competitors and *trans*-competitors with other *trans*-competitors. This yields two possible comparisons: 3'*trans *versus 5'*trans *and 5'*cis *versus 3'*cis, *see Figure 2, with which we can measure the effects of co-transcriptional folding.

We proceed as follows to detect if co-transcriptional folding takes place: For every RNA sequence of the data set, we detect events of type 3'*cis, 3'trans, 5'cis *and *5'trans*, where an alternative helix competes with a known helix. Each such event is given two different weights, see Table 1 for an overview of definitions: (1) a weight of 1/ (*d·log*(*l*)), where *d *is the distance between the two competing helices and *l *is the length of the sub-sequence 5' or 3' of the known helix on which the competing helix falls, or (2) a weight of |*G*| / (*d*·log(*l*)), where the former weight is multiplied by the absolute value of the free energy *G *of the competing, alternative helix. The factor 1/*d *gives alternative helices that are far away from the known helix a smaller weight than closer ones. The factor 1*/log (l) *accounts for the fact that *log (l) *is proportional to the expected sum of 1*/d *statistics for a sub-sequence of length *l *(i.e. the integral ). The free energy factor *G *in the second type of weights gives stable alternative helices which have a larger impact on the folding pathway a greater weight than helices which are easily unfolded. Statistics derived from weights of type 1*/(d *log(*l*)) are denoted by an index *p *(for plain) and those of type |*G*| / (*d*·log(*l*)) by an index *g *(for free energy). By summing the weighted counts for each of the four classes of events, we thus arrive at eight different scalar values which characterize each RNA sequence: *3'Trans*_*x*_*, 3'Cis*_*x*_*, 5'Trans*_*x *_and *5'Cis*_*x *_for *x *∈ {*p,g*}.

**Table 1:** Definitions of the different statistics. Definitions of the different statistics used. *i *and  denote the sequence positions of a base-pair in the known structure, *c *is an alternative pairing partner for *i *(but according to the base-pairing rules therefore not for ), *L *is the length of the RNA sequence, *N *is the number of sequences in the data set and the index *x *indicates the type of weight used. Please refer to the text for a description of how alternative pairing partners are calculated.

*x*	*p *plain weights	*g *free energy weights
*3'cis*_*x*_	1/((*c *- *i*) log(*L *- *i*))	|*G*_*ci*_|/((*c *- *i*) log(*L*-*i*))
*3'trans*_*x*_	1/((*c *- ) log(*L *- ))	|*G*_*ci*_|/((c - ) log(*L *- ))
5'*cis*_*x*_	1/((*i*-*c*) log(*i*))	|G_*ic*_|/((*i*-*c*) log(*i*))
5'*trans*_*x*_	1/(( - *c*)log())	|G_*ic*_|/(( - *c*) log())
*cis*_*x*_	5'*cis*_*x *_- 3'*cis*_*x*_	
*trans*_*x*_	3'*trans*_*x *_- 5'*trans*_*x*_	
3'*Cis*_*x*_	Σ_#3'*cis *_3'*cis*_*x*_	
3'*Trans*_*x*_	Σ_#3'*trans *_3'*trans*_*x*_	
5'*Cis*_*x*_	Σ_#5'*cis *_5'*cis*_*x*_	
5'*Trans*_*x*_	Σ_#5'*trans *_5'*trans*_*x*_	
*Cis*_*x*_	5'*Cis*_*x *_- 3'*Cis*_*x*_	
*Trans*_*x*_	3'*Trans*_*x *_- 5'*Trans*_*x*_	
		
where *x *∈ {*p,g*}, *y *∈ {3'*Cis*, 3'*Trans*, 5'*Cis*, 5'*Trans*, *Cis*, *Trans*}		

We can now define the two statistics which are capable of measuring the two main types of asymmetry within each RNA sequence:

*Cis *:= 5'*Cis *- 3'*Cis*

*Trans *:= 3'*Trans *- 5'*Trans*

which can calculate for both types of weights. Without co-transcriptional folding, the expectation value of these two statistics is zero. Co-transcriptional folding induces two types of asymmetries by suppressing the number of alternative helices which compete with the final helices (indicated by an increased number of  configurations, see Figure 2) and by promoting the formation of transient helices which guide the correct folding (indicated by an increased number of  configurations). Both types of effects are indicated by an expectation value larger than zero for the respective statistics.

Without co-transcriptional folding, the introduced statistics have an expectation of zero, moreover, the distributions should be symmetric. The number of positive cases *(pos) *thus follows a binomial distribution with parameter *p = *0.5 and the statistic



where *n *is the number of all cases, approximately follows a standard normal distribution. If this value is sufficiently positive, we have to reject the hypothesis that co-transcriptional folding is not encoded within RNA genes.

### Data

All 16S rRNA, 23S rRNA as well as Group I and Group II type intron sequences with completely known secondary structures were downloaded from the Comparative RNA Web (CRW) Site [35,36], resulting in 304 16S rRNA, 84 23S rRNA, 15 Group I intron and 6 Group II intron sequences from three main taxonomical units (Archea, Bacteria, Eukaryotes) and two organelles, see Table 2.

**Table 2:** Composition of the two data sets.

Taxonomic unit	all	16S rRNA	23S rRNA	Group I	Group II
Data set A					

Archea	28	22	6	0	0
Bacteria	277	232	45	0	0
Eukaryotes	41	35	6	0	0
Chloroplasts	6	6	0	0	0
Mitochondria	9	9	0	0	0
Sum	361	304	57	0	0
Data set B					
Eukaryotes	15	0	0	15	0
Bacteria	5	0	5	0	0
Chloroplasts	5	0	5	0	0
Mitochondria	23	0	17	0	6
Sum	48	0	27	15	6

Organellar 23S rRNA sequences frequently contain Group I introns and recent research revealed that the 23S rRNA of several hyperthermophilic bacteria also have Group I intron [37]. Other species only rarely have introns in rRNA genes, however, some 16S rRNA introns are known [38].

rRNA genes in bacteria are encoded in the so-called rrn-operon (see for example [39]). The canonical order of rRNA genes in the rrn-operon is 16S-23S-5S, but some exceptions to this rule are known. In *Vibrio harvey, *the order is 23S-16S-5S [40], but not in *Vibrio cholerae *[41] and *Vibrio parahaemolyticus *[42], whose 16S rRNA sequences were downloaded from the Comparative RNA Web Site.

We divided the gathered sequences into two sets: data set A which consists of all RNA sequences that are thought to correspond to the originally transcribed sequence units and data set B which contains all those RNA sequences that are known to differ from the originally transcribed sequence units. Data set B thus contains the Group I and II intron sequences, organellar and hyperthermophilic bacteria 23S RNA sequences. As we neither know the sequence nor the secondary structure of the original transcript units from which the sequences of data set B were derived, we are limited to detecting the effects of co-transcriptional folding within these shorter sequences. We expect this to be much more difficult than in sequences that correspond to the originally transcribed sequence units as co-transcriptional folding introduces long range effects which are harder to detect the shorter the investigated sub-sequence gets. See Table 2 for a detailed overview of the composition of each data set.

## Results

**Figure 3:**
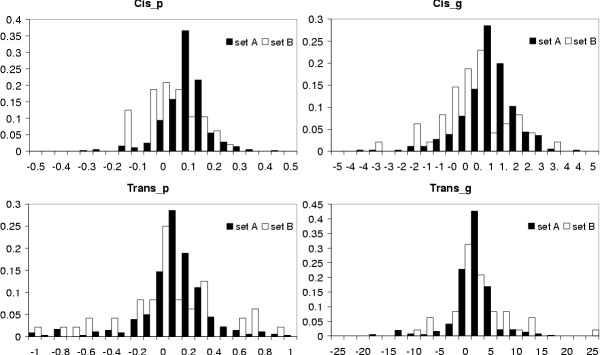
**Distribution of *Cis *and *Trans *values. **Distribution of *Cis *and *Trans *values for the sequences of data sets A and B and both types of weights (plain (p) or free energy based (g)). The area under each curve has been normalized to one to allow a direct comparison between the two data sets.

We calculated the *3'Cis*_*x*_*, 3'Trans*_*x*_*, 5'Cis*_*x *_and *5'Trans*_*x *_values for both types of weights, i.e. *x *∈ {*p,g*}, for each sequence in the two data sets. From these values we then derived each sequence's *Cis*_*x *_and *Trans*_*x *_values, again for both *x *types of weights. Their distributions are shown in Figure 3. Averaging over the values of all sequences in each of the two data sets resulted in the final values shown in Table 3.

**Table 3:** Average values for different statistics. Final values of the different statistics which were obtained by averaging the values of each sequence in the data set. The error shown is the standard deviation.

dataset						
A	0.215 ± 0.009	0.461 ± 0.032	0.285 ± 0.009	0.382 ± 0.032	0.070 ± 0.004	0.079 ± 0.026
B	0.298 ± 0.040	0.562 ± 0.086	0.296 ± 0.043	0.521 ± 0.075	-0.003 ± 0.015	0.041 ± 0.082
dataset						
A	2.916 ± 0.106	6.236 ± 0.431	3.710 ± 0.111	5.134 ± 0.354	0.794 ± 0.061	1.102 ± 0.384
B	3.392 ± 0.406	7.033 ± 1.050	3.362 ± 0.456	6.380 ± 0.954	-0.030 ± 0.184	0.653 ± 1.253

The first thing to note in Figure 3 is that all distributions follow approximately a symmetric distribution, thus confirming our theoretical considerations, and that the distributions of data set B are always shifted towards lower values with respect to the corresponding distributions for data set A which are always centered around average values larger than zero.

The mean values of *Cis *and *Trans *in Table 3 are positive for data set A for both types of weights, indicating the influence of co-transcriptional folding, whereas they are closer to zero or even negative in the case of data set B.

A *Cis *value larger zero means that configurations of type  outnumber those of type , see Figure 2. The formation of potential transient helices involving base-pairs between *c *and *i *that can later yield to the final secondary structure element containing the base-pair between *i *and  thus seems to be encouraged. However, these transient structure elements may not be too stable if they are to guide rather than impede the proper folding. The presence of transient helices could thus be further substantiated by showing that these transient helices are less stable than the final helix. In contrast to the  configuration, the competing *ic *helices in the  case are suppressed as they lie 3' of the final  helix and thus emerge later in time during co-transcriptional folding. A *Cis *value larger than zero can therefore be explained by the presence of temporary helices which may guide the formation of the final, functional secondary structure during co-transcriptional folding.

A *Trans *value larger than zero means that  configurations are less frequent than  configurations, see Figure 2. In the  configuration, both *c *and  are competing pairing partners for *i *as they both emerge before *i *during transcription. This may lead to the formation of wrong *ci *helices, whereas the order of pairing partners in the  configuration has a lower risk of mis-folding due the *c *emerging only after the  and thus only after the  helix could have already formed.

In addition, *3'Trans > 3'Cis *in Table 3 can be interpreted as a stabilization of the final, functional secondary structure. Imagine that the hydrogen bounds of the  or  helix temporarily break up. In the case of the *3'Trans *configuration, the pairing partners come in the order  along the RNA sequence, whereas they come in the order  in the *3'Cis *configuration. In the  order, the *c *part is in vicinity to the *i *part, so the possibility of ending up with a wrong refolding due to a *ic *helix is larger than in the  case.

Overall, we can thus conclude from the average values in Table 3, that the sequences of data set A are tailored towards co-transcriptional folding, whereas we cannot reliably detect the effects of co-transcriptional folding within data set B. We detected co-transcriptional folding in data set A by showing that the final secondary structure is actively stabilized *(3'Trans > 3'Cis), *that the formation of temporary helices may guide the structure formation and that these helices may thus be used to actively engineer a folding pathway *(Cis >*0) and that secondary structure elements which may interfere with the formation of the final, functional secondary structure during co-transcriptional folding are suppressed *(Trans >*0).

In order to quantify the influence of co-transcriptional folding further, we calculated two statistics, a t-test for the hypothesis that the given statistics have an expectation value of zero as well as the p-value of the number of positive cases for our two co-transcriptional folding indicators, see Table 4. The high p-values for data set B imply that the presence of co-transcriptional folding is not well supported in this data set. However, the corresponding indicators strongly support co-transcriptional folding within data set A.

**Table 4:** Statistical significance of results. p-values of t-test for the hypothesis that the final values in Table 3 have an expectation value of zero as well as the p-values for the hypothesis that the number of positive cases follows a binomial distribution with parameter 0.5.

dataset	A		B	
	p-value for t-test	p-value for *pos*	p-value for t-test	p-value for *pos*
	< 0.0001	< 0.0001	0.5733	0.6137
	< 0.0001	< 0.0001	0.5650	0.6137
	0.0012	< 0.0001	0.3093	0.8068
	0.0021	< 0.0001	0.3011	0.5000

## Discussion

Recent experimental studies [23,24,19] have shown that the proper speed of transcription helps the correct folding of RNA molecules. In addition, theoretical studies [16] indicate that the functional structure of an RNA need not correspond to the minimum free energy structure, even for moderately long RNA molecules. These findings suggest that co-transcriptional folding may play a decisive role in the formation of functional RNA structures.

Although our statistics are able to reveal two general effects of co-transcriptional folding within data set A, we cannot conclude that they would be powerful enough to serve as a reliable indicator of co-transcriptional folding for single RNA sequences, as some of the sequences in data set A may not correspond to the originally transcribed sequence units. In addition, all of our statistics consider only a first order effect of co-transcriptional folding by studying alternative helices for the known helices, but do not take higher order effects into account as e.g. alternative helices of alternative helices etc.

Based on computer simulations, H. Isambert et. al. [43] conjecture that pseudo-knotted motifs are common in co-transcriptional folding. Pseudo-knotted structures are explicitly included in our statistics, as the corresponding calculations naturally allow for alternative helices which are part of a pseudo-knot and as we do not reject them.

## Conclusions

To summarize, our findings show that co-transcriptional folding is a guiding principle in the formation of functional RNA structure and that it can influence both the primary and potential secondary structures of an RNA molecule. This has several implications. Current algorithms for RNA secondary structure prediction can probably be improved by adopting co-transcriptional folding as a guiding principle rather than only free energy minimization. This may hopefully provide the extra information needed to be able to reliably detect RNA genes [44]. Several groups have already come up with computer algorithms which attempt to fold an RNA sequence co-transcriptionally [45-48,22]. These findings also have implications for computational methods which infer the phylogeny of RNA sequences, as these consider only co-evolution within the base-pairs of the functional helices, but discard any information due to the conservation of folding pathways and may hence mis-estimate evolutionary times. Similar arguments hold for all comparative studies that aim to detect functional secondary structure elements, since co-evolution of nucleic acids does not necessarily imply that these nucleic acids are base-paired in the final functional secondary structure. As evolution probably not only selects for the correct functional secondary structure, but also for a suitable folding pathway, it should be possible to detect the effects of co-transcriptional folding also in a comparative way.

Most importantly, co-transcriptional folding should lead to a better understanding of *how *RNA sequences fold. This should in turn enable us to also understand why some RNA sequences mis-fold and fail to function properly in the organism. Even though protein folding is known to differ in many respects from RNA folding, they also have some features in common [49]. One of the obvious similarities is that both proteins and RNA sequences are synthesized in a directional process. It would thus be interesting to investigate if protein folding is also influenced by *co-translational folding.*

In this study, we neither attempted to study the effects that co-transcriptional folding may have on sequences that are transcribed together (e.g. genes in an operon) nor to study the influence that the binding by proteins or RNA sequences or RNA editing may have on the co-transcriptional folding pathway and the final, functional RNA structure. This will almost certainly require more refined investigation methods, but we hope that this study provides enough insight and motivation to start to tackle these exciting questions.

## Authors' contributions

I.M.M. proposed this work and contributed the main idea for the statistics. I.M. selected the data and evaluated the statistical significance of the results. Both authors shared the programming tasks and the writing of the manuscript.

## References

[B1] Mathews DH, Sabina J, Zuker M, Turner DH (1999). Expanded sequence dependence of thermodynamic parameters improves prediction of RNA secondary structure. J Mol Biol.

[B2] Zuker M (2000). Calculating nucleic acid secondary structure. Curr Opin Struct Biol.

[B3] Zuker M (2003). Mfold web server for nucleic acid folding and hybridization prediction. Nucleic Acids Res.

[B4] Zuker M, Stiegler P (1981). Optimal computer folding of large RNA sequences using thermodynamic and auxiliary information. Nucleic Acids Res.

[B5] Hofacker I, Fontana W, Stadler P, Bonhoeffer S, Tacker M, Schuster P (1994). Fast Folding and Comparison of RNA Secondary Strutures. Monatsh Chem (Chem Monthly).

[B6] Wuchty S, Fontana W, Hofacker I, Schuster P (1999). Complete Suboptimal Folding of RNA and the Stability of Secondary Structures. Biopolymers.

[B7] Hofacker I, Fekete M, Stadler P (2002). Secondary Structure Prediction for Aligned RNA Sequences. J Mol Biol.

[B8] Hofacker I (2003). The Vienna RNA Secondary Structure Server. Nucleic Acids Res.

[B9] Eddy SR, Durbin R (1994). RNA sequence analysis using covariance models. Nucleic Acids Res.

[B10] Lowe T, Eddy S (1997). tRNAscan-SE: a Program For Improved Detection of Transfer RNA genes in Genomic Sequence. Nucleic Acids Res.

[B11] Knudsen B, Hein J (1999). RNA Secondary Structure Prediction Using Stochastic Context-Free Grammars and Evolutionary History. Bioinformatics.

[B12] Knudsen B, Hein J (2003). Pfold: RNA secondary structure prediction using stochastic context-free grammars. Nucleic Acids Res.

[B13] Rivas E, Eddy SR (2001). Noncoding RNA gene detection using comparative sequence analysis. BMC Bioinformatics.

[B14] Flamm C, Hofacker I, Stadler P (1999). RNA In Silico: The Computational Biology of RNA Secondary Structures. Adv Complex Systems.

[B15] Flamm C, Fontana W, Hofacker I, Schuster P (2000). RNA folding at elementary step resolution. RNA.

[B16] Morgan S, Higgs P (1996). Evidence for kinetic effects in the folding of large RNA molecules. J Chem Phys.

[B17] Boyle J, Robillard G, Kim S (1980). Sequential folding of transfer RNA. A nuclear magnetic resonance study of successively longer tRNA fragments with a common 5' end. J Mol Biol.

[B18] Kramer F, Mills D (1981). Secondary structure formation during RNA-synthesis. Nucleic Acids Res.

[B19] Repsilber D, Wiese S, Rachen M, Schroder A, Riesner D, Steger G (1999). Formation of metastable RNA structures by sequential folding during transcription: Time-resolved structural analysis of potato spindle tuber viroid (-)-stranded RNA by temperature-gradient gel electrophoresis. RNA.

[B20] Ro-Choi T, Choi Y (2003). Structural elements of dynamic RNA strings. Molecules and Cells.

[B21] Harlepp S, Marchal T, Robert J, Leger J, Xayaphoummine A, Isambert H, Chatenay D (2003). Probing complex RNA structures by mechanical force. Eur Phys J E.

[B22] Isambert H, Siggia E (2000). Modeling RNA folding paths with pseudoknots: Application to hepatitis delta virus ribozyme. Proc Natl Acad Sci USA.

[B23] Lewicki B, Margus T, Remme J, Nierhaus K (1993). Coupling of rRNA transcription and ribosomal assembly in vivo – formation of active ribosomal-subunits in Esccherichia coli requires transcription of RNA genes by host RNA polymerase which cannot be replaced by T7 RNA polymerase. J Mol Biol.

[B24] Chao MY, Kan M, Lin-Chao S (1995). RNAII transcribed by IPTG-induced T7 RNA polymerase is non-functional as a replication primer for ColEl-type plasmids in Escherichia coli. Nucleic Acids Res.

[B25] Heilmann-Miller SL, Woodson SA (2003). Effect of transcription on folding of the *Tetrahymena *ribozyme. RNA.

[B26] Proudfoot N, Furger A, Dye M (2002). Integrating rnRNA processing with transcription. Cell.

[B27] Neugebauer K (2002). On the importance of being co-transcriptional. J Cell Sci.

[B28] Herschlag D (1995). RNA chaperones and the RNA folding problem. J Biol Chem.

[B29] Turner D, Sugimoto N, Freier S (1990). Nucleic Acids, Springer Verlag Thermodynamics and kinetics of base-pairing and of DNA and RNA self-assembly and helix coil transition.

[B30] Mohr Gand Zhang A, Gianelos J, Belfort M, Lambowitz A (1992). The neurospora CYT-18 protein suppresses defects in the phage T4 td intron by stabilizing the catalytically active structure of the intron core. Cell.

[B31] Mueller F, Brimacombe R (1997). A new model for the three-dimensional folding of Escherichia coli 16 S ribosomal RNA. 2. The RNA-protein interaction data. J Mol Biol.

[B32] Farina K, Singer R (2002). The nuclear connection in RNA transport and localization. Trends Cell Biol.

[B33] Balzer M, Wagner R (1998). Mutations in the leader region of ribosomal RNA operons cause structurally defective 30 S ribosomes as revealed by in vivo structural probing. J Mol Biol.

[B34] Besancon W, Wagner R (1999). Characterization of transient RNA-RNA interactions important for the facilitated structure formation of bacterial ribosomal 16S RNA. Nucleic Acids Res.

[B35] Cannone J, Subramanian S, Schnare M, Collett J, D'Souza L, Du Y, Feng B, Lin N, Madabusi L, Muller K, Pande N, Shang Z, Yu N, Gutell R (2002). The Comparative RNA Web (CRW) Site: an online database of comparative sequence and structure information for ribosomal, intron, and other RNAs. BMC Bioinformatics.

[B36] Cannone J, Subramanian S, Schnare M, Collett J, D'Souza L, Du Y, Feng B, Lin N, Madabusi L, Muller K, Pande N, Shang Z, Yu N, Gutell R (2002). The Comparative RNA Web (CRW) Site: an online database of comparative sequence and structure information for ribosomal, intron, and other RNAs: Correction. BMC Bioinformatics.

[B37] Nesbo C, Doolittle W (2003). Active self-splicing group I introns in 23S rRNA genes of hyperthermophilic bacteria, derived from introns in eukaryotic organelles. Proc Natl Acad Sci USA.

[B38] Itoh T, Nomura N, Sako Y (2003). Distribution of 16S rRNA introns among the family Thermoproteaceae and their evolutionary implications. Extremophiles.

[B39] Martín JF, Barreiro C, Gonzalez-Lavado E, Barriuso M (2003). Ribosomal RNA and ribosomal proteins in corynebacteria. J Biotech.

[B40] Lamfrom H, Sarabhai A, Abelson J (1978). Cloning of beneckea genes in *Escherichia *coli. J Bacterial.

[B41] Heidelberg J, Eisen J, Nelson W, RA C, Gwinn M, Dodson R, Haft D, Hickey E, Peterson J, Umayam L, Gill S, Nelson K, TD R, Tettelin H, Richardson D, Ermolaeva M, Vamathevan J, Bass S, Qin H, Dragoi I, Sellers P, McDonald L, Utterback T, Fleishmann R, Nierman W, White O, Salzberg S, Smith H, Colwell R, Mekalanos J, Venter J, Fraser C (2000). DNA sequence of both chromosomes of the cholera pathogen Vibrio cholerae. Nature.

[B42] Makino K, Oshima K, Kurokawa K, Yokoyama K, Uda T, Tagomori K, Iijima Y, Najima M, Nakano M, Yamashita A, Kubota Y, Kimura S, Yasunaga T, Honda T, Shinagawa H, Hattori M, Iida T (2003). Genome sequence of Vibrio parahaemolyticus: a pathogenic mechanism distinct from that of V cholerae. Lancet.

[B43] Xayaphoummine A, Bucher T, Thalmann F, Isambert H (2003). Prediction and statistics of pseudoknots in RNA structures using exactly clustered stochastic simulations. Proc Natl Acad Sci USA.

[B44] Rivas E, Eddy SR (2000). Secondary structure alone is generally not statistically significant for the detection of noncoding RNAs. Bioinformatics.

[B45] Mironov A, Dyakonova L, Kister A (1985). A kinetic approach to the prediction of RNA secondary structures. J Biomol Struct Dyn.

[B46] Mironov A, Kister A (1986). RNA secondary structure formation during transcription. J Biomol Struct Dyn.

[B47] Gultyaev A (1991). The computer-simulation of RNA folding involving pseudoknot formation. Nucleic Acids Res.

[B48] Gultyaev A, von Batenburg F, Pleij C (1995). The computer-simulation of RNA folding pathways using a genetic algorithm. J Mol Biol.

[B49] Thirumalai A, Woodson S (1996). Kinetics of Folding of Proteins and RNA. Acc Chem Res.

